# Utility of the Z-score of log-transformed A Body Shape Index (LBSIZ) in the assessment for sarcopenic obesity and cardiovascular disease risk in the United States

**DOI:** 10.1038/s41598-019-45717-8

**Published:** 2019-06-26

**Authors:** Wankyo Chung, Jung Hwan Park, Hye Soo Chung, Jae Myung Yu, Dong Sun Kim, Shinje Moon

**Affiliations:** 1 Department of Public Health Science, Graduate School of Public Health, Seoul National University, Seoul, Republic of Korea; 2 Institute of Health and Environment, Seoul National University, Seoul, Republic of Korea; 3 Department of Internal Medicine, Hanyang University College of Medicine, Seoul, Republic of Korea; 4 Division of Endocrinology and Metabolism, Hallym University College of Medicine, Chuncheon, Republic of Korea; 5 Department of Internal medicine, Graduate School, Hanyang University, Seoul, Republic of Korea

**Keywords:** Metabolic syndrome, Obesity

## Abstract

Body mass index (BMI) has limited accuracy for predicting cardiovascular diseases (CVD) and is not capable of identifying sarcopenic obesity, the combination of sarcopenia (an age-associated decline in muscle mass and physical function) and obesity. To overcome this, the z-score of the log-transformed A Body Shape Index (LBSIZ) was recently introduced as a measure of obesity using waist circumference, height, and weight. We aimed to investigate the association of LBSIZ with sarcopenic obesity and CVD, and propose appropriate cut-off values using the National Health and Nutrition Examination Survey 1999–2016 data. Of 92,062 participants, 40,468 adults (≥20 years) were included. Overall area under curve (AUC) of LBSIZ was 0.735 (95% confidence interval [CI]: 0.716–0.754) for sarcopenic obesity, and 0.695 (95% CI: 0.687–0.703) for CVD. The subgroup analysis of ethnicity/race showed similar results. Waist circumference (WC), BMI, conicity index, body roundness index (BRI), Clinica Universidad de Navarra-Body Adiposity Estimator (CUN-BAE), new BMI, and waist to height ratio (WHtR) showed a negative association with sarcopenic obesity, while LBSIZ and conicity index showed a positive association. The AUC of LBSIZ was significantly higher for sarcopenic obesity than that of conicity index (p < 0.001). The AUC of LBSIZ was significantly higher for CVD than those of parameters including WC, BMI, BRI, CUN-BAE, new BMI, and WHtR (p < 0.001). The AUC for conicity index alone was comparable to that of LBSIZ for CVD. Overall LBSIZ cut-off was 0.35 for both sarcopenic obesity (sensitivity, 65.3%; specificity, 71.5%) and CVD (sensitivity, 63.3%; specificity, 66.6%). These results may be useful not only to identify sarcopenic obesity, but also to conduct CVD risk assessment in the clinical setting.

## Introduction

According to the World Health Organization (WHO), 13% of the world population were estimated to be obese in 2016, the prevalence of which increased three times in the last three decades^1^. Obesity increases the risk of chronic diseases, such as cardiovascular disease (CVD), diabetes, stroke, and cancer, which are responsible for approximately 4.8% of worldwide deaths^2–6^. The increasing prevalence of obesity and serious obesity-related diseases has made obesity a major public health concern. Another concern with aging population is sarcopenic obesity, which is the combined state of sarcopenia and obesity^7^. Sarcopenia is defined as the age-associated decline in muscle mass and physical function, and may synergistically worsen the adverse effects of obesity, leading to higher disability, morbidity and mortality^7^.

Accurate assessment of obesity is required for the prevention and treatment thereof. Obesity can be assessed by directly measuring body fat via computed tomography (CT), magnetic resonance imaging, DEXA, and positron emission tomography (PET)-CT^8^. However, these methods are costly and have limitations that make the use of these modalities for diagnosing obesity in real clinical settings challenging; instead, indirect indices of obesity are used. Body mass index (BMI) has long been used, as it is easy to measure and calculate. However, BMI has limited accuracy for predicting the amount and distribution of body fat and is not capable of identifying sarcopenic obesity^9^. It is also limited in its ability to clinically predict the risk of chronic diseases such as CVD^10–12^. To overcome these limitations, A Body Shape Index (ABSI), which is a formula that uses waist circumference (WC), height, and weight, has recently been introduced^13^. However, ABSI has limited clinical usefulness due to not having cut-off points for identifying individuals at high risk for obesity-related diseases^14,15^. Therefore, we have previously proposed the z-score of the log-transformed ABSI (LBSIZ), which overcomes the limitations of the ABSI, using representative samples from Korea^15–17^. However, data is lacking in terms of its assessed usefulness in other populations.

Therefore, this study aimed to propose a LBSIZ formula for each race, using representative samples from the United States (US). We then examined its relationship with both sarcopenic obesity and CVD risk, compared to other obesity parameters, and provided appropriate cut-off values to identify individuals at high risk for sarcopenic obesity and CVD.

## Results

### Baseline characteristics

**Figure 1:**
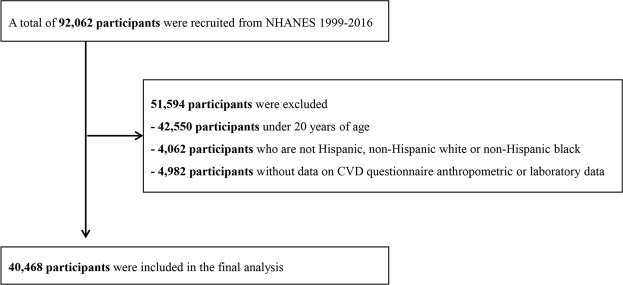
Flowchart showing the final selection. NHANES, National Health and Nutrition Examination Survey; CVD, cardiovascular disease.

A total of 40,468 healthy adults (19,508 men and 20,960 women) from the US, aged 20–85 years (mean age = 49.2), were analyzed (Fig. 1). The anthropometric, clinical, and biochemical characteristics of the participants are summarized in Table 1 according to ethnicity/race. A total of 4,327 (10.7%) participants had CVD (angina pectoris: 2.9%; coronary heart disease: 4.1%; myocardial infarction: 4.2%; congestive heart failure: 3.1%; cerebrovascular disease: 3.4%). The distributions of LBSIZ according to ethnicity/race are summarized in Table 2.

**Table 1:** Characteristics of the subjects according to ethnicity/race.

Variable	Ethnicity/race				Total
	Hispanic (N = 11596)	Non-Hispanic White (N = 19815)	Non-Hispanic Black (N = 9057)	*P*	
Age (years)	46.3 ± 16.9	51.6 ± 19.0	47.7 ± 17.0	<0.001	49.2 ± 18.1
Men	5423 (46.8%)	9722 (49.1%)	4363 (48.2%)	<0.001	19508 (48.2%)
Smoking (≥100 cigarettes in life)	4539 (39.2%)	10522 (53.1%)	3960 (43.8%)	<0.001	19021 (47.0%)
BMI (kg/m^2^)	29.3 ± 6.0	28.3 ± 6.4	30.2 ± 7.5	0.002	29.0 ± 6.6
Waist circumference (cm)	98.5 ± 14.2	99.1 ± 16.1	99.8 ± 17.3	<0.001	99.1 ± 15.9
LBSIZ	−0.01 ± 1.01	−0.01 ± 1.00	0.00 ± 1.00	0.736	−0.01 ± 1.00
Systolic BP (mmHg)	122.6 ± 19.7	123.6 ± 18.9	127.2 ± 20.8	<0.001	124.1 ± 19.7
Diastolic BP (mmHg)	69.2 ± 12.7	69.7 ± 12.8	71.6 ± 14.6	<0.001	70.0 ± 13.2
Hypertension	4135 (38.2%)	8947 (47.4%)	4665 (54.6%)	<0.001	17747 (46.4%)
FBG level (mg/dL)	111.1 ± 41.9	105.0 ± 29.7	108.4 ± 42.2	<0.001	107.5 ± 36.5
HbA_1c_ (%)	5.8 ± 1.3	5.5 ± 0.8	5.9 ± 1.3	<0.001	5.7 ± 1.1
Diabetes Mellitus	2175 (19.3%)	2636 (13.6%)	1795 (21.0%)	<0.001	6606 (16.9%)
Total cholesterol (mg/dL)	198.9 ± 42.0	198.8 ± 43.3	192.0 ± 41.9	<0.001	197.4 ± 42.7
TG (mg/dL)	165.9 ± 150.2	150.5 ± 130.0	110.3 ± 83.8	<0.001	146.4 ± 129.8
HDL-C (mg/dL)	50.4 ± 14.4	53.3 ± 16.6	56.1 ± 17.2	<0.001	53.0 ± 16.3
Dyslipidemia	4458 (52.9%)	9070 (54.6%)	3373 (48.3%)	<0.001	16901 (52.8%)
CVD	837 (7.2%)	2548 (12.9%)	942 (10.4%)	<0.001	4327 (10.7%)

Data are presented as the means ± SD or number (%).

Abbreviations: CVD, cardiovascular disease; BMI, body mass index; LBSIZ, z-score of the log-transformed A Body Shape Index; WC, waist circumference; BP, blood pressure; FBG, fasting blood glucose; HbA1c, haemoglobin A1c; HDL-C, high-density lipoprotein cholesterol; TG, triglyceride.

**Table 2:** Distribution of LBSIZ according to ethnicity/race.

Ethnicity/race	Percentile								
	10^th^	20^th^	30^th^	40^th^	50^th^	60^th^	70^th^	80^th^	90^th^
Total	−1.30	−0.84	−0.52	−0.24	0.02	0.26	0.53	0.84	1.26
Hispanic	−1.28	−0.83	−0.50	−0.23	0.01	0.24	0.51	0.81	1.25
Non-Hispanic White	−1.31	−0.84	−0.53	−0.25	0.01	0.27	0.54	0.85	1.27
Non-Hispanic Black	−1.30	−0.85	−0.52	−0.24	0.04	0.29	0.55	0.85	1.26

LBSIZ, z-score of the log-transformed A Body Shape Index.

### Association between obesity parameters and body composition

**Figure 2:**
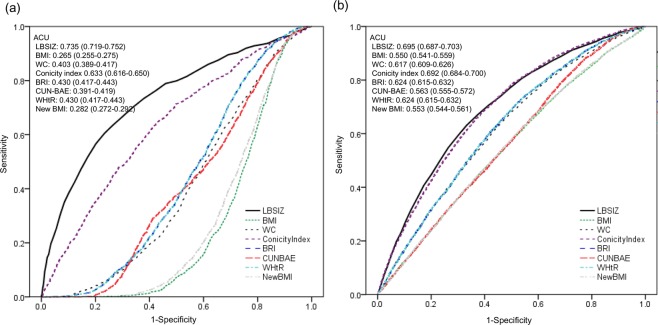
Receiver operating characteristic curves for CVD according to obesity parameters. (**a**) Sarcopenic obesity; (**b**) CVD. LBSIZ: z-score of the log-transformed A Body Shape Index; BMI: body mass index; WC: waist circumference; Body Roundness Index: BRI; Clinica Universidad de Navarra-Body Adiposity Estimator: CUN-BAE; waist to height ratio: WHtR; CVD: cardiovascular disease.

**Figure 3:**
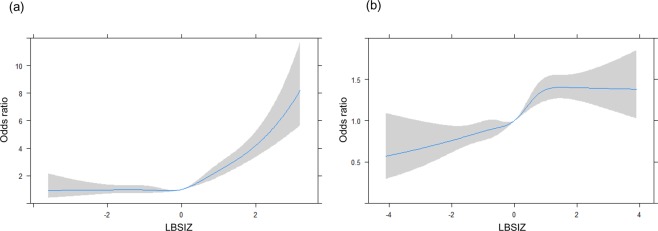
The odds ratio according to the continuous value of LBSIZ. (**a**) Sarcopenic obesity; (**b**) CVD. Adjusted for age, sex and ethnicity/race. LBSIZ: z-score of the log-transformed A Body Shape Index (LBSIZ).

LBSIZ showed a positive association with fat mass index (FMI) and a negative association with appendicular skeletal mass index (ASMI), while all other obesity parameters showed a positive association with both FMI and ASMI (Table 3). Figure 2a shows the ROC curves for sarcopenic obesity according to obesity parameters. The overall Area Under the Curve (AUC) of LBSIZ for sarcopenic obesity was 0.735 (95% confidence interval [CI]: 0.716–0.754). Other obesity parameters showed a negative association with sarcopenic obesity, while LBSIZ and conicity index showed a positive association. The AUC of LBSIZ was significantly higher than that of conicity index (p < 0.001). In the subgroup analysis according to ethnicity/race, the AUCs for sarcopenic obesity were 0.717 (95% CI: 0.681–0.753), 0.740 (95% CI: 0.716–0.764), and 0.804 (95% CI: 0.733–0.876) for the Hispanic, Non-Hispanic white, and Non-Hispanic black groups, respectively. The overall cut-off value of LBSIZ for sarcopenic obesity was 0.35 (65^th^ percentile, sensitivity, 65.3%; specificity, 71.5%). The corresponding sensitivity and specificities by ethnicity/race were 65.1% and 70.9%, 64% and 62.3%, and 81.8% and 60.6% for the Hispanic, Non-Hispanic white, and Non-Hispanic black groups, respectively. The OR for sarcopenic obesity adjusted for age, sex, and ethnicity/race was 2.38 (95% CI, 2.05–2.77) at the estimated cut-off value of LBSIZ. On the restricted cubic spline regression plot, ORs increased after the median value (0) of LBSIZ (Fig. 3b)

**Table 3:** Association of obesity parameters with FMI and ASMI.

	FMI	ASMI
	*β* (95% CI)	*β* (95% CI)
LBSIZ	0.178 (0.112, 0.245)	−0.221 (−0.244, −0.198)
WC	0.223 (0.221, 0.225)	0.060 (0.059, 0.061)
BMI	0.602 (0.598, 0.606)	0.184 (0.182, 0.186)
Conicity Index	21.075 (20.434, 21.717)	3.244 (2.989, 3.499)
BRI	1.720 (1.703, 1.736)	0.454 (0.445, 0.463)
CUN-BAE	0.467 (0.463, 0.0.470)	0.146 (0.144, 0.148)
New BMI	0.586 (0.583, 0.590)	0.178 (0.176, 0.180)
WHtR	37.794 (37.440, 38.148)	10.036 (9.836, 10.236)

Linear regression analysis adjusted for age, sex and ethnicity/race.

LBSIZ: z-score of the log-transformed A Body Shape Index; BMI: body mass index; WC: waist circumference; Body Roundness Index: BRI; Clinica Universidad de Navarra-Body Adiposity Estimator: CUN-BAE; waist to height ratio: WHtR.

### Association between obesity parameters and CVD

Figure 2b shows the ROC curves for CVD according to the obesity parameters. The overall AUC of LBSIZ for CVD was 0.695 (95% CI: 0.687–0.703), which was significantly higher than those of WC (p value < 0.001), BMI (p value < 0.001), Body Roundness Index (BRI) (p < 0.001), Clinica Universidad de Navarra-Body Adiposity Estimator (CUN-BAE) (p < 0.001), New BMI (p < 0.001) and waist to height ratio (WHtR) (p < 0.001). Conicity index alone showed a comparable AUC to that of LBSIZ. The bootstrap’s bias corrected 95% CI of AUC of LBSIZ was 0.687–0.703. In the subgroup analysis according to ethnicity/race, the AUCs of LBSIZ were 0.680 (95% CI: 0.661–0.699), 0.708 (95% CI: 0.697–0.718), and 0.678 (95% CI: 0.661–0.695) for the Hispanic, Non-Hispanic white, and Non-Hispanic black groups, respectively. The overall cut-off value of LBSIZ was 0.35 (65^th^ percentile, sensitivity, 63.3%; specificity, 66.6%). Its corresponding sensitivity and specificities by ethnicity/race were 62.4% and 66.2%, 64.6% and 67.4%, and 60.7% and 65.3% for the Hispanic, Non-Hispanic white, and Non-Hispanic black groups, respectively. The OR for CVD, after adjusting for age, sex, and ethnicity/race was 1.40 (95% CI, 1.30–1.51) at the estimated cut-off value of LBSIZ. On the restricted cubic spline regression plot, ORs increased after the median (0) of LBSIZ (Fig. 3b).

## Discussion

This study investigated the association of LBSIZ with both sarcopenic obesity and CVD, using a representative US sample, and found that LBISZ showed superior association thereto, compared with other weight-, and WC-related obesity measures. The study also provided appropriate cut-off values of LBSIZ, to be able to identify individuals at high risk for sarcopenic obesity or CVD, irrespective of sex and race, and to improve its clinical usefulness in practice.

A number of epidemiologic studies showed heterogeneous results regarding the association between traditional BMI and CVD^10–12^. This heterogeneity might be due to the limitations in defining obesity based on BMI, which does not differentiate fat from lean mass, nor does it consider the distribution of adipose tissue^1,18–25^. As central deposition of adipose tissues due to obesity became known to be a major cause of CVD-related mortality and morbidity, WC has emerged as an important complement to BMI, as an indicator of visceral adiposity, metabolic risk, and increased morbidity and mortality^26–30^. The National Cholesterol Education Program–Adult Treatment Panel III (NCEP–ATP III) criteria use WC instead of BMI to define metabolic syndrome^31^. However, there are insufficient data regarding the appropriate WC values to define obesity among the different age groups and sexes^19,28,32^. Due to these limitations of BMI and WC, many researchers have explored other obesity indices^33,34^.

In 2012, Krakauer *et al*. proposed a new obesity index, ABSI^12^, using the WC, weight, and height data from the NHANES 1999–2004. Several studies reported that ABSI predicted premature mortality and CVD more effectively than did BMI or WC^13,35,36^. However, other studies reported that the role of ABSI has been challenged as a risk predictor of mortality, cardiovascular diseases, and metabolic syndrome^32,37,38^. A recent meta-analysis of 38 studies reported ambiguous results, where ABSI outperformed BMI and WC in predicting all-cause mortality, but underperformed in predicting hypertension, and diabetes mellitus^39^. In addition, several studies reported that ABSI had limitations in predicting fat mass^40^.

Although LBSIZ is a revised measure of abdominal obesity based on ABSI, it was better in predicting the development of CVD, than BMI or WC, and even improved the predictability of the Framingham risk score for CVD events in a prospective Korean cohort^15^. In another population-based study using the Korea NHANES data, LBSIZ showed a linear relationship with CVD^17,41^. Interestingly, this study showed that LBISZ has a superior association with CVD in a representative US sample, compared with other obesity parameters. Although the mechanism is unclear, we suspect that the association of obesity parameters with body composition might have played an important intermediary role thereto. In this regard, therefore, the result of LBSIZ having a positive association with FMI, and a negative association with ASMI, is consistent with the results of the previous Rotterdam study^14^. Conversely, other obesity parameters, associated positively with both fat mass and ASMI, could not identify sarcopenic obesity, or the presence of low muscle mass accompanied by a high fat mass^42^.

A complex (albeit not fully elucidated) interplay of several underlying mechanisms are responsible for the development of sarcopenic obesity; fat accumulation, which is related to the increase of proinflammatory cytokines, oxidative stress, and insulin resistance, might cause muscle fiber atrophy and mitochondrial dysfunction, leading to the development and progression of sarcopenia. Likewise, sarcopenia could worsen obesity through the decline of physical activity and energy expenditure, thereby resulting in further sarcopenia, leading to a vicious cycle of atrophy, aggravating their effects on metabolic, and functional abnormalities^43,44^. While a clear definition of sarcopenic obesity was not available, several studies showed that it was related to metabolic diseases and physical disability^45–47^; a few studies about the relationship between sarcopenic obesity and CVD, and mortality have been performed^48–50^. According to a recent meta-analysis, sarcopenic obesity was assessed to be associated with a 24% increased risk of all-cause mortality^51^. This study used Baumgartner’s definition of sarcopenic obesity^42^, and showed that LBSIZ is the only measure of obesity related to sarcopenic obesity among all the obesity parameters that were considered. These results indicate the usefulness of LBSIZ in screening sarcopenic obesity, unlike other obesity measures.

LBSIZ has limitations in its use in clinical settings, or in epidemiological studies, considering the calculation of LBSIZ is highly complicated. Therefore, we present a simpler formula that can be used to estimate LBSIZ for each race, using a large-scale dataset from the NHANES 1999–2016. In addition, the estimated cut-off value can be used as a clinical standard, thereby facilitating easier clinical use, irrespective of sex or race. Considering the increase in ORs for sarcopenic obesity and CVD after the median LBSIZ in the restricted cubic spline regression plots, the cut-off value corresponding to the 65^th^ percentile of LBSIZ can effectively help to identify individuals at a high risk of sarcopenic obesity and CVD.

Despite our interesting findings, there are a few limitations in this study. First, this was a cross-sectional study, and therefore, the causal relationship between obesity and CVD remains to be examined further, using cohort data. Secondly, this study did not analyze mortality, and may have missed fatal CVD events due to a lack of data. Third, we only examined weight-, and WC-related obesity measures, omitting hip circumference-related measures due to data availability. Finally, potential confounders are yet to be further examined to elucidate the LBSIZ-related pathophysiological mechanism.

In conclusion, LBSIZ showed a stronger association with both sarcopenic obesity and CVD, compared with other obesity parameters. These results may be useful when conducting a CVD risk assessment in clinical settings; the proposed LBSIZ cut-off values have the potential to be a useful clinical standard.

## Materials and Methods

### Study population

Data were collected from the NHANES dataset between 1999 and 2016. Exclusion criteria were as follows: those aged ≤20 years, those with missing data (CVD questionnaire, anthropometric, or laboratory data), or those who were not Hispanic, non-Hispanic whites, or non-Hispanic blacks. Finally, 40,468 of 92,062 participants were included in this study (Fig. 1). Because the DEXA data were available between 1999 and 2005, 11,780 participants were included in the subgroup analysis for body composition.

### Measurements of obesity parameters and body composition

WC was measured using a measuring tape at the upper-lateral border of the iliac crest^52^. BMI was defined by the weight in kilograms, divided by the height in meters squared (kg/m^2^). Conicity Index, BRI, CUN-BAE, new BMI, and WHtR were calculated based on the earlier-suggested formula^13,32,53,54^^.^

$${\rm{Conicity}}\,{\rm{Index}}:{\rm{WC}}({\rm{m}})/[0.1093\,{\rm{sqrt}}({\rm{Weight}}({\rm{kg}})/{\rm{Height}}({\rm{m}}))]$$

$${\rm{BRI}}:364.2-365.5\times {\rm{sqrt}}[1-({({\rm{WC}}/(2{\rm{\pi }}))}^{2}/{(0.5\times {\rm{Height}})}^{2})]$$

$$\begin{array}{c}{\rm{CUN}} \mbox{-} {\rm{BAE}}:\mbox{--}\,44.988+(0.503\times {\rm{age}})+(10.689\times {\rm{sex}})+(3.172\times {\rm{BMI}})\\ \,-\,(0.026\times {\rm{BMI}}2)+(0.181\times {\rm{BMI}}\times {\rm{sex}})-(0.02\times {\rm{BMI}}\times {\rm{age}})\\ \,-\,(0.005\times {\rm{BMI}}2\times {\rm{sex}})+(0.00021\times {\rm{BMI}}2\times {\rm{age}})\\ \,{\rm{where}}\,{\rm{male}}=0\,{\rm{and}}\,{\rm{female}}=1\,{\rm{for}}\,{\rm{sex}}\end{array}$$

$${\rm{New}}\,{\rm{BMI}}:1.3\times ({\rm{Weight}}({\rm{kg}})/{\rm{Height}}{({\rm{m}})}^{2})$$

$${\rm{WHtR}}:{\rm{WC}}({\rm{cm}})/{\rm{Height}}({\rm{cm}})$$

We calculated the LBSIZ based on the regression [ln(waist) = a_0_ + a_1_ln(weight) + a_2_ln(height) + δ], standardizing the waist values according to weight and height. After respective estimation for each race, the log-transformed ABSI (LBSI) was calculated using the following equation: log [waist/(exp(a_0_) × weight^a1^ × height^a2^]. In the next step, the z-score of the log-transformed ABSI (LBSIZ) was calculated using the mean of the LBSI and standard deviation (SD) of LBSI: LBSIZ = (LBSI-mean(LBSI))/SD(LBSI). More details are provided in the supplementary Excel file, and are referenced in a previous study^16^.

Assessment of body composition was performed by whole body DEXA using a Hologic QDR 4500 A fan beam X-ray bone densitometer (Hologic Inc., Marlborough, MA, USA). All DEXA scans were analyzed using Hologic Discovery software (version 12.1, Hologic Inc.) to measure total and regional body composition, including bone mineral content lean body mass, fat mass, and % body fat. ASM was defined as the sum of the total lean mass of both the arms and legs. ASM index was defined as ASM divided by the square of the height. Fat mass index was defined as the total fat mass divided by the square of the height.

### Definition of sarcopenic obesity and CVD

Sarcopenic obesity was defined as ASMI < 7.26 kg/m^2^, and % body fat >27% in men, or ASMI < 5.45 kg/m^2^, and % body fat >38% in women, based on the definitions by Baumgartner^42^

A structured questionnaire was used to investigate CVD. A patient was deemed to have CVD if they had at least one of the following conditions: angina pectoris, coronary heart disease, myocardial infarction, congestive heart failure, or cerebrovascular disease^55^.

### Statistical analysis

For summary statistics, we presented the mean with a 95% CI, or prevalence (%) according to ethnicity/race. Continuous variables were assessed using one-way analysis of variance, and categorical variables were assessed using the Pearson’s chi-square test. We estimated the distribution of LBSIZ, and verified the correlations between the obesity indices. A receiver operating characteristic (ROC) curve was used to analyze the correlation between sarcopenic obesity or CVD, and each of the obesity parameters, and the de Long’s test was used to identify obesity indices that were significantly superior^56^. We validated the model using 1,000 samples generated through the bootstrap method. The cut-off value of LBSIZ was determined as the value with the highest Youden’s index, conditional on having a sensitivity and specificity greater than 60%^57^. A multivariate logistic regression analysis was performed to determine the OR for sarcopenic obesity and CVD. Furthermore, the OR of LBSIZ for sarcopenic obesity and CVD was analyzed using restricted cubic spline splits with five knots. The analyses were performed using SPSS software version 24.0 (IBM Inc., Armonk, NY, USA), R (version 3.1.0, R Foundation for Statistical Computing, Vienna, Austria) and Stata version 15 (Stata Corporation, College Station, Texas, USA). A P-value of 0.05 was considered statistically significant.

### Ethics statement

The study protocol was approved by the institutional review board of Kangnam Sacred Heart Hospital (IRB No. HKS 2017-07-007). All participants volunteered, and provided written informed consent prior to their enrolment. All participants’ records were anonymized before being accessed by the authors. All methods were carried out in accordance with the approved guidelines and regulations.

## Supplementary information

Supplementary Dataset 1
